# Clinicopathological Parameters and Biomarker Profile in a Cohort of Patients With Head and Neck Squamous Cell Carcinoma (HNSCC)

**DOI:** 10.7759/cureus.41941

**Published:** 2023-07-15

**Authors:** Atif A Hashmi, Ummara Bukhari, Mahnoor Aslam, Rana Sajawal Joiya, Ravi Kumar, Umair Arshad Malik, Shamail Zia, Abdur Rahim Khan, Mubasshir Saleem, Muhammad Irfan

**Affiliations:** 1 Pathology, Liaquat National Hospital and Medical College, Karachi, PAK; 2 Internal Medicine, Jinnah Sindh Medical University, Karachi, PAK; 3 Internal Medicine, Baqai Medical University, Karachi, PAK; 4 Public Health Sciences, University of Alberta, Edmonton, CAN; 5 Pathology, CMH Lahore Medical College and Institute of Dentistry, Lahore, PAK; 6 Internal Medicine, Chandka Medical College, Larkana, PAK; 7 Internal Medicine, Aga Khan University, Karachi, PAK; 8 Pathology, Jinnah Sindh Medical University, Karachi, PAK; 9 Pathology, Karachi Medical and Dental College, Karachi, PAK; 10 Internal Medicine, University of Alberta, Edmonton, CAN; 11 Dermatology, Dermcare Institute of Canada, Edmonton, CAN; 12 Statistics, Liaquat National Hospital and Medical College, Karachi, PAK

**Keywords:** p53, egfr, clinicopathological parameters, p27, p16, biomarkers, squamous cell carcinoma, head and neck cancer

## Abstract

Introduction: Squamous cell carcinoma (SCC) is the most common malignancy of the head and neck region, commonly termed as head and neck squamous cell carcinoma (HNSCC). Data related to biomarker expression in HNSCC are scarcely available, especially in our population. This study aimed to evaluate the association of immunohistochemical (IHC) expression of p16, epidermal growth factor receptor (EGFR), p27, and p53 in HNSCC with clinical and pathological parameters.

Methods: This retrospective cross-sectional study was conducted at the Department of Histopathology, Liaquat National Hospital, Karachi, Pakistan from February 2017 to January 2022. A total of 308 cases of HNSCC with upfront surgical resection were included in the study. IHC analysis was performed for EGFR, p16, p27, and p53, and association with clinicopathological parameters was sought.

Results: p16, EGFR, and p53 positivity were noted in 22.1%, 18.8%, and 66.2% cases, respectively, whereas loss of p27 expression was seen in 14.3% cases of HNSCC. A significant association of p16 expression was observed with age, tumor size, tumor site, nodal metastasis, extranodal extension (ENE), and perineural invasion (PNI). Cases aged over 50 years were more significantly associated with positive p16. Similarly, cases with oral cavity SCC were more significantly associated with positive p16. HNSCC with larger tumor size, the presence of nodal metastasis, and ENE and PNI were associated with negative p16 expression. Similarly, a significant association of EGFR expression was observed with age, tumor size, tumor site, histological subtype, histological differentiation, nodal metastasis, ENE, and PNI (p < 0.05). Cases of HNSCC with age less than 50 years were associated with positive EGFR expression. Similarly, oral cavity and lip SCCs were associated with positive EGFR expression compared with other sites. Moreover, positive EGFR expression was significantly associated with nodal metastasis, ENE, moderate histological differentiation, and the presence of PNI. Loss of p27 expression was significantly associated with nodal stage and ENE; low nodal stage and absence of ENE were associated with p27 loss of expression, whereas no significant association was seen with other pathological parameters. Alternatively, a significant association of mutant-type p53 expression was noted with gender, nodal stage, and histological subtype. Females with HNSCC show a higher frequency of mutant-type p53 expression than males. Moreover, higher nodal stage (N2b and higher) and non-keratinizing SCCs were significantly associated with mutant-type p53 expression.

Conclusion: Our study found a high expression of EGFR and mutant-type p53 expression in HNSCC. Conversely, p16 expression and loss of p27 expression were low. Moreover, EGFR and mutant-type p53 expression were associated with poor pathological parameters, whereas p16 expression was associated with better histological features.

## Introduction

Squamous cell carcinoma (SCC) is the most common malignancy of the head and neck region, commonly termed as head and neck squamous cell carcinoma (HNSCC) [1-2]. Human papilloma virus (HPV) oral infection is one of the most important risk factors for the occurrence of oral SCC, followed by smoking and alcohol consumption [3]. Areca nut usage is emerging as another important risk factor for oral SCC. The incidence of HNSCC in Southeast Asian countries is on the rise owing to the use of areca nuts (pan/gutka) [4]. Opposed to HPV-induced SCC in Western countries, Southeast Asian HNSCCs are more aggressive with a high recurrence rate [5].

Metastasis of neck nodes is one of the most important prognostic factors in HNSCC. Other prognostic pathological parameters include tumor size, depth of invasion (DOI), lymphovascular invasion (LVI), and perineural invasion (PNI). Despite the high mortality of HNSCC, the role of targeted therapy is not yet established, nor is the significance of prognostic biomarkers. p16 and p53 are tumor suppressor genes found in human cells. Mutations in these genes lead to the formation of truncated proteins that are resistant to protease digestion and are therefore abnormally expressed. Conversely, sometimes null mutations of p53 also lead to a complete absence of p53 protein, as detected by immunohistochemical (IHC) analysis [6-8]. The high expression of p16 is also associated with HPV infection [9-10]. Similarly, the loss of p27 expression was also associated with HNSCC [4].

Epidermal growth factor receptor (EGFR) is a proto-oncogene; high expression of EGFR was found in many human cancers and is associated with a poor prognosis [11-13]. There is a scarcity of data related to biomarker expression in HNSCC, especially in our population. This study aimed to evaluate the association of p16, EGFR, p27, and p53 expression in HNSCC with clinical and pathological parameters.

## Materials and methods

This retrospective cross-sectional study was conducted at the Department of Histopathology, Liaquat National Hospital, Karachi, Pakistan from February 2017 to January 2022, over a period of five years. All cases were biopsy-proven cases of HNSCCs. The cases in which upfront surgical resection of HNSCC with neck dissection was performed were included in the study. Cases with locally advanced disease that hindered upfront surgery or distant metastasis at the time of diagnosis were excluded from the study. Similarly, cases that had neoadjuvant chemotherapy or radiation before surgery were also excluded from the study.

All specimens were received in the histopathology laboratory, followed by a gross examination. The tumor size was grossly noted, and representative sections from the tumor were submitted for microscopic examination. Similarly, lymph nodes were dissected from the neck dissection specimen and submitted for microscopic examination. Sections from the bone were submitted after decalcification.

Microscopic examination was performed by senior histopathologists. Histopathological features such as tumor grade/differentiation were recorded along with DOI and PNI. The number of lymph nodes involved by the metastatic tumor was recorded along with the presence of extranodal extension (ENE). Histopathological typing was performed based on the proportion of keratinization. Tumors with less than 10% keratinization were termed as non-keratinizing, 10%-30% keratinization as non-keratinizing with maturation, and more than 30% keratinization as keratinizing SCC. Grading was done based on architecture (including keratinization) and cytological atypia. Grade 1/well-differentiated SCC was keratinizing with mild atypia; Grade 2/moderately differentiated had moderate keratinization and atypia, while Grade 3/poorly differentiated had severe atypia with minimal keratinization. DOI was measured from the level of adjacent normal epithelium to the deepest level of tissue invasion. The exophytic portion of tumors was excluded from the DOI measurement.

Immunohistochemical analysis

The IHC analysis was performed on the representative tissue blocks according to standard protocols. p16 was taken as positive if more than 70% of tumor cells exhibited strong positive nuclear and cytoplasmic expression. p27 was categorized as loss of expression if more than 80% of tumor cells showed loss of normal nuclear expression. Adjacent normal squamous epithelial cells showed nuclear expression; used positive controls. p53 was called a mutant-type expression if more than 80% of tumor cells exhibited strong nuclear expression. EGFR was taken as positive if more than 30% of tumor cells labeled strong membranous expression.

Statistical analysis

Data analysis was performed using IBM SPSS Statistics for Windows, Version 26.0 (Released 2019; IBM Corp., Armonk, New York). The means were calculated for patient age and DOI, whereas frequencies and percentages were calculated for all other clinicopathological variables and biomarker expression. A p-value of <0.05 was considered significant. Chi-square and Fisher’s exact tests were applied to determine the association of clinicopathological features with IHC expression of EGFR, p16, p27, and p53.

## Results

A total of 308 cases of HNSCC were included in the study. The mean age at diagnosis was 51.51 ± 12.35 years with a male predominance (76%). Most tumors at the time of definite resection were between 2.1 and 4 cm in size, with a mean DOI of 1.176 ± 0.77 cm. The most common site of the occurrence of HNSCC was the oral cavity (66.9%). Nodal metastasis was observed in 45.5% of the cases, and the most common histological subtype was keratinizing SCC (59.1%) with moderate differentiation (64.3%). PNI was present in a small proportion of cases (13%). p16, EGFR and p53 positivity was noted in 22.1%, 18.8%, and 66.2% cases, respectively, whereas loss of p27 expression was seen in 14.3% cases of HNSCC, as shown in Table 1.

**Table 1:** Clinicopathological parameters of the study population (n=308). SD, standard deviation; N, nodal; EGFR, epidermal growth factor receptor

Clinicopathological parameters	Values
Gender	
Male, n (%)	234 (76)
Female, n (%)	74 (24)
Age (years), mean ± SD	51.51 ± 12.35
Age groups	
≤30 years, n (%)	16 (5.2)
31-50 years, n (%)	150 (48.7)
>50 years, n (%)	142 (46.1)
Tumor size (cm), mean ± SD	3.35±1.72
Tumor size groups	
≤2 cm, n (%)	68 (22.1)
2.1-4.0 cm, n (%)	156 (50.6)
>4 cm, n (%)	84 (27.3)
Depth of invasion (cm), mean ± SD	1.176 ± 0.77
Depth of invasion groups	
≤1 cm, n (%)	152 (49.4)
>1 cm, n (%)	156 (50.6)
Tumor site	
Oral cavity, n (%)	206 (66.9)
Lip, n (%)	12 (3.9)
Tongue, n (%)	74 (24)
Soft palate, n (%)	16 (5.2)
Nodal metastasis	
Present, n (%)	140 (45.5)
Absent, n (%)	168 (54.5)
N stage	
N0, n (%)	168 (54.5)
N1, n (%)	38 (12.3)
N2b, n (%)	94 (30.5)
N2c, n (%)	4 (1.3)
N3, n (%)	4 (1.3)
Extranodal extension	
Present, n (%)	78 (25.3)
Absent, n (%)	230 (74.7)
Histological subtype	
Non-keratinizing, n (%)	38 (12.3)
Keratinizing, n (%)	182 (59.1)
Non-keratinizing with maturation, n (%)	88 (28.6)
Histological differentiation/Grade	
Well-differentiated/Grade 1, n (%)	82 (26.6)
Moderately differentiated/Grade 2, n (%)	198 (64.3)
Poorly differentiated/Grade 3, n (%)	28 (9.1)
Perineural invasion	
Present, n (%)	40 (13)
Absent, n (%)	268 (87)
p16	
Positive, n (%)	68 (22.1)
Negative, n (%)	240 (77.9)
EGFR	
Positive, n (%)	58 (18.8)
Negative, n (%)	250 (81.2)
p27	
No loss of expression, n (%)	264 (85.7)
Loss of expression, n (%)	44 (14.3)
p53	
Mutant-type, n (%)	204 (66.2)
Wild-type, n (%)	104 (33.8)

Table 2 shows the association of p16 expression with clinical and pathological parameters in HNSCC. A significant association of p16 expression was observed with age, tumor size, tumor site, nodal metastasis, ENE, and PNI. Cases aged over 50 years were more significantly associated with positive p16 (52.9%). Similarly, cases with oral cavity SCC were more significantly associated with positive p16 (76.5%), whereas tongue SCC was less likely to be associated with positive p16. HNSCC with larger tumor size, the presence of nodal metastasis, and ENE and PNI were associated with negative p16 expression.

**Table 2:** Association of p16 expression with clinicopathological parameters in HNSCC. N, nodal; HNSCC, head and neck squamous cell carcinoma *Chi-square test was applied. **Fisher’s exact test was applied. ***p-value significant as < 0.05.

Clinicopathological parameters	Values		p-value
	p16		
	Positive	Negative	
Gender*			
Male, n (%)	52 (76.5)	182 (75.8)	0.914
Female, n (%)	16 (23.5)	58 (24.2)	
Age groups**			
≤30 years, n (%)	0 (0)	16 (6.7)	0.044***
31-50 years, n (%)	32 (47.1)	118 (49.2)	
>50 years, n (%)	36 (52.9)	106 (44.2)	
Tumor size groups*			
≤2 cm, n (%)	16 (23.5)	52 (21.7)	<0.001***
2.1-4.0 cm, n (%)	48 (70.6)	108 (45)	
>4 cm, n (%)	4 (5.9)	80 (33.3)	
Depth of invasion groups*			
≤1 cm, n (%)	32 (47.1)	120 (50)	0.668
>1 cm, n (%)	36 (52.9)	120 (50)	
Site**			
Oral cavity, n (%)	52 (76.5)	154 (64.2)	0.031***
Lip, n (%)	4 (5.9)	8 (3.3)	
Tongue, n (%)	8 (11.8)	66 (27.5)	
Soft palate, n (%)	4 (5.9)	12 (5)	
Nodal metastasis*			
Present, n (%)	16 (23.5)	124 (51.7)	<0.001***
Absent, n (%)	52 (76.5)	116 (48.3)	
N stage**			
N0, n (%)	52 (76.5)	116 (48.3)	<0.001***
N1, n (%)	0 (0)	38 (15.8)	
N2b, n (%)	12 (17.6)	82 (34.2)	
N2c, n (%)	4 (5.9)	0 (0)	
N3, n (%)	0 (0)	4 (1.7)	
Extranodal extension*			
Present, n (%)	4 (5.9)	74 (30.8)	<0.001***
Absent, n (%)	64 (94.1)	166 (69.2)	
Histological subtype*			
Non-keratinizing, n (%)	8 (11.8)	30 (12.5)	0.060
Keratinizing, n (%)	48 (70.6)	134 (55.8)	
Non-keratinzing with maturation, n (%)	12 (17.6)	76 (31.7)	
Histological differentiation/Grade*			
Well-differentiated/Grade 1, n (%)	24 (35.3)	58 (24.2)	0.145
Moderately differentiated/Grade 2, n (%)	40 (58.8)	158 (65.8)	
Poorly differentiated/Grade 3, n (%)	4 (5.9)	24 (10)	
Perineural invasion*			
Present	4 (5.9)	36 (15)	0.048***
Absent	64 (94.1)	204 (85)	

Table 3 depicts the association of EGFR expression in HNSCC with clinicopathological features. A significant association of EGFR expression was observed with age, tumor size, tumor site, histological subtype, histological differentiation, nodal metastasis, ENE, and PNI (p < 0.05). Cases of HNSCC with age less than 50 years were associated with positive EGFR expression (69%). Similarly, oral cavity and lip SCCs were associated with positive EGFR expression compared with other sites. Moreover, positive EGFR expression was significantly associated with nodal metastasis, ENE, moderate histological differentiation, and the presence of PNI.

**Table 3:** Association of EGFR expression with clinicopathological parameters in HNSCC. EGFR, epidermal growth factor receptor; N, nodal; HNSCC, head and neck squamous cell carcinoma *Chi-square test was applied. **Fisher’s exact test was applied. ***p-value significant as < 0.05.

Clinicopathological parameters	Values		p-Value
	EGFR		
	Positive	Negative	
Gender*			
Male, n (%)	46 (79.3)	188 (75.2)	0.509
Female, n (%)	12 (20.7)	62 (24.8)	
Age groups**			
≤30 years, n (%)	8 (13.8)	8 (3.2)	0.001***
31-50 years, n (%)	32 (55.2)	118 (47.2)	
>50 years, n (%)	18 (31)	124 (49.6)	
Tumor size groups*			
≤2 cm, n (%)	4 (6.9)	64 (25.6)	<0.001***
2.1-4.0 cm, n (%)	18 (31)	138 (55.2)	
>4 cm, n (%)	36 (62.1)	48 (19.2)	
Depth of invasion groups*			
≤1 cm, n (%)	26 (44.8)	126 (50.4)	0.444
>1 cm, n (%)	32 (55.2)	124 (49.6)	
Site**			
Oral cavity, n (%)	46 (79.3)	160 (64)	0.010***
Lip, n (%)	4 (6.9)	8 (3.2)	
Tongue, n (%)	8 (13.8)	66 (26.4)	
Soft palate, n (%)	0 (0)	16 (6.4)	
Nodal metastasis*			
Present, n (%)	38 (65.5)	102 (40.8)	0.001***
Absent, n (%)	20 (34.5)	148 (59.2)	
N stage**			
N0, n (%)	20 (34.5)	148 (59.2)	0.004***
N1, n (%)	10 (17.2)	28 (11.2)	
N2b, n (%)	28 (48.3)	66 (26.4)	
N2c, n (%)	0 (0)	4 (1.6)	
N3, n (%)	0 (0)	4 (1.6)	
Extranodal extension*			
Present, n (%)	26 (44.8)	52 (20.8)	<0.001***
Absent, n (%)	32 (55.2)	198 (79.2)	
Histological subtype*			
Non-keratinizing, n (%)	16 (27.6)	22 (8.8)	<0.001***
Keratinizing, n (%)	22 (37.9)	160 (64)	
Non-keratinizing with maturation, n (%)	20 (34.5)	68 (27.2)	
Histological differentiation/grade*			
Well-differentiated/Grade 1, n (%)	2 (3.4)	80 (32)	<0.001***
Moderately differentiated/Grade 2, n (%)	52 (89.7)	146 (58.4)	
Poorly differentiated/Grade 3, n (%)	4 (6.9)	24 (9.6)	
Perineural invasion*			
Present, n (%)	16 (27.6)	24 (9.6)	<0.001***
Absent, n (%)	42 (72.4)	226 (90.4)	

Table 4 presents the association of p27 expression with clinicopathological parameters. Loss of p27 expression was significantly associated with nodal stage and ENE; low nodal stage and absence of ENE were associated with p27 loss of expression, whereas no significant association was seen with other pathological parameters.

**Table 4:** Association of p27 expression with clinicopathological parameters in HNSCC. N, nodal; HNSCC, head and neck squamous cell carcinoma *Chi-square test was applied. **Fisher’s exact test was applied. ***p-value significant as < 0.05.

Clinicopathological parameters	Values		p-Value
	p27		
	No loss of expression (n=264)	Loss of expression (n=44)	
Gender*			
Male, n (%)	198 (75)	36 (81.8)	0.327
Female, n (%)	66 (25)	8 (18.2)	
Age groups**			
≤30 years, n (%)	12 (4.5)	4 (9.1)	0.406
31-50 years, n (%)	130 (49.2)	20 (45.45)	
>50 years, n (%)	122 (46.2)	20 (45.45)	
Tumor size groups*			
≤2 cm, n (%)	56 (21.2)	12 (27.3)	0.119
2.1-4.0 cm, n (%)	140 (53)	16 (36.4)	
>4 cm, n (%)	68 (25.8)	16 (36.4)	
Depth of invasion groups*			
≤1 cm, n (%)	132 (50)	20 (45.5)	0.577
>1 cm, n (%)	132 (50)	24 (54.5)	
Site**			
Oral cavity, n (%)	178 (67.4)	28 (63.6)	0.082
Lip, n (%)	8 (3)	4 (9.1)	
Tongue, n (%)	62 (23.5)	12 (27.3)	
Soft palate, n (%)	16 (6.1)	0 (0)	
Nodal metastasis*			
Present, n (%)	124 (47)	16 (36.4)	0.191
Absent, n (%)	140 (53)	28 (63.6)	
N stage**			
N0, n (%)	140 (53)	28 (63.6)	0.001*
N1, n (%)	34 (12.9)	4 (9.1)	
N2b, n (%)	86 (32.6)	8 (18.2)	
N2c, n (%)	0 (0)	4 (9.1)	
N3, n (%)	4 (1.5)	0 (0)	
Extranodal extension*			
Present, n (%)	74 (28)	4 (9.1)	0.007***
Absent, n (%)	190 (72)	40 (90.9)	
Histological subtype*			
Non-keratinizing, n (%)	30 (11.4)	8 (18.2)	0.442
Keratinizing, n (%)	158 (59.8)	24 (54.5)	
Non-keratinizing with maturation, n (%)	76 (28.8)	12 (27.3)	
Histological differentiation/Grade**			
Well-differentiated/Grade 1, n (%)	74 (28)	8 (18.2)	0.388
Moderately differentiated/Grade 2, n (%)	166 (62.9)	32 (72.7)	
Poorly differentiated/Grade 3, n (%)	24 (9.1)	4 (9.1)	
Perineural invasion*			
Present, n (%)	32 (12.1)	36 (81.8)	0.268
Absent, n (%)	232 (87.9)	8 (18.2)	

Table 5 shows the association of p53 with clinicopathological parameters. A significant association of mutant-type p53 expression was noted with gender, nodal stage, and histological subtype. Females with HNSCC show a higher frequency of mutant-type p53 expression than males. Moreover, higher nodal stage (N2b and higher) and non-keratinizing SCCs were significantly associated with mutant-type p53 expression. Conversely, no significant expression was noted with respect to age, tumor size, DOI, tumor site, grade, ENE, and PNI. 

**Table 5:** Association of p53 expression with clinicopathological parameters in HNSCC. N, nodal; HNSCC, head and neck squamous cell carcinoma *Chi-square test was applied. **p-value significant as < 0.05. ***Fisher’s exact test was applied.

Clinicopahological parameters	Values		p-Value
	p53		
	Mutant-type (n=204)	Wild-type (n=104)	
Gender*			
Male, n (%)	134 (65.7)	100 (96.2)	<0.001**
Female, n (%)	70 (34.3)	4 (3.8)	
Age groups*			
≤30 years, n (%)	8 (3.9)	8 (7.7)	0.297
31-50 years, n (%)	98 (48)	52 (50)	
>50 years, n (%)	98 (48)	44 (42.3)	
Tumor size groups*			
≤2 cm, n (%)	48 (23.5)	20 (19.2)	0.209
2.1-4.0 cm, n (%)	96 (47.1)	60 (57.7)	
>4 cm, n (%)	60 (29.4)	24 (23.1)	
Depth of invasion groups*			
≤1 cm, n (%)	104 (51)	48 (46.2)	0.423
>1 cm, n (%)	100 (49)	56 (53.8)	
Site*			
Oral cavity, n (%)	134 (65.7)	72 (69.2)	0.883
Lip, n (%)	8 (3.9)	4 (3.8)	
Tongue, n (%)	50 (24.5)	24 (23.1)	
Soft palate, n (%)	12 (5.9)	4 (3.8)	
Nodal metastasis*			
Present, n (%)	100 (49)	40 (38.5)	0.078
Absent, n (%)	104 (51)	64 (61.5)	
N stage***			
N0, n (%)	104 (51)	64 (61.54)	0.001**
N1, n (%)	18 (8.81)	20 (19.23)	
N2b, n (%)	74 (36.27)	20 (19.23)	
N2c, n (%)	4 (1.96)	0 (0)	
N3, n (%)	4 (1.96)	0 (0)	
Extranodal extension*			
Present, n (%)	54 (26.5)	24 (23.1)	0.517
Absent, n (%)	150 (73.5)	80 (76.9)	
Histological subtype*			
Non-keratinizing, n (%)	34 (16.67)	4 (3.8)	0.005**
Keratinizing, n (%)	114 (55.88)	69 (65.4)	
Non-keratinizing with maturation, n (%)	56 (27.45)	32 (30.8)	
Histological differentiation/Grade*			
Well-differentiated/Grade 1, n (%)	46 (22.5)	36 (34.6)	0.076
Moderately differentiated/Grade 2, n (%)	138 (67.6)	60 (57.7)	
Poorly differentiated/Grade 3, n (%)	20 (9.8)	8 (7.7)	
Perineural invasion*			
Present, n (%)	28 (13.7)	12 (11.5)	0.589
Absent, n (%)	176 (86.3)	92 (88.5)	

## Discussion

In this study, we evaluated the biomarker expression in HNSCC. We found high expression of EGFR and p53 (mutant-type), whereas p16 expression and p27 loss of expression were relatively low. Moreover, EGFR expression was associated with poor prognostic parameters, such as nodal metastasis, ENE, and PNI. Similarly, p53 mutant-type expression was associated with a higher nodal stage. Conversely, p16 expression was associated with better prognostic parameters, such as smaller tumor size, lower nodal stage, and lack of PNI. In a similar context, the loss of p27 expression was related to the lower nodal stage.

We noted a high expression of EGFR in HNSCC in our study. Verma et al. [14] studied EGFR expression in 48 cases of HNSCC. In their study, 25% of cases showed high EGFR expression, whereas 60% of cases revealed intermediate EFGR expression. They noted an association of EGFR expression with histological grade, whereas no significant association was noted with nodal metastasis, as seen in our study.

Mutant-type p53 expression was observed in 66.2% of HNSCC cases in our cohort of cases, with a significant association with higher nodal stage and non-keratinizing subtype. In accordance with these findings. Kakkar et al. [15] in a study involving 100 cases of oral SCC concluded that high p53 expression was associated with poor overall survival and disease-free survival in HNSCC.

We noted a relatively low expression of p16 in our cases of HNSCC, signifying a low prevalence of HPV oral infection in our population. We found that p16-expressing cancers in our study had better histological parameters, such as smaller tumor size and lack of nodal metastasis and PNI. Concordant with these findings, p16 expression showed a survival benefit in oral SCC [16].

The proliferation and growth of cancer cells are associated with the up-and-down-regulation of various cell cycle pathways. Down-regulation of the cyclin-dependent kinase (CDK) inhibitor p27 is a well-recognized pathway of carcinogenesis in HNSCC [17]. Data on p27 association with pathological parameters are conflicting. A meta-analysis suggested that low p27 expression was associated with higher stage and nodal metastasis [18]; however, such an association was not seen in our study.

Limitations

First, our study had a few limitations, the most important of which is that we did not follow our patients to evaluate the association of biomarker expression in HNSCC with overall survival and recurrence-free survival, as recurrence in HNSCC is of utmost importance, prognostically. Second, HNSCC has many risk factors, such as smoking, alcohol, HPV infection, and areca-nut chewing. We did not evaluate the association of risk factors with biomarker expression in HNSCC. Evaluating the association of biomarker expression with risk factors may help in devising national preventive protocols. Finally, the association of IHC expression with molecular alterations was not sought in our study.

## Conclusions

Our study provided an association of histological and clinical parameters with biomarker profiles in HNSCC. In this study, we noted a high expression of EGFR and p53 (mutant-type) in HNSCC, whereas positive p16 expression and loss of p27 expression were relatively low. Moreover, EGFR and p53 mutant-type expression were significantly associated with poor histological parameters, such as nodal metastasis. Alternatively, p16 expression was associated with better prognostic parameters, such as the lack of PNI and lower nodal stage. Additionally, the histological subtype was significantly associated with biomarker expression. Non-keratinizing subtype was associated with EGFR and mutant p53 expression, whereas p16 expression was associated with keratinizing subtype.

## References

[1] Johnson DE, Burtness B, Leemans CR (2020). Head and neck squamous cell carcinoma. Nat Rev Dis Primers.

[2] Hashmi A A, Tola R, Rashid K (2023). Clinicopathological parameters predicting nodal metastasis in head and neck squamous cell carcinoma. Cureus.

[3] Mehanna H, Beech T, Nicholson T (2013). Prevalence of human papillomavirus in oropharyngeal and nonoropharyngeal head and neck cancer--systematic review and meta-analysis of trends by time and region. Head Neck.

[4] Hashmi AA, Aijaz S, Irfan M (2019). Low p27kip1 expression in head and neck squamous cell carcinoma: association with risk factors and adverse outcomes. Appl Cancer Res.

[5] Hashmi AA, Iftikhar SN, Haider R (2020). Recurrence and disease-free survival in head and neck squamous cell carcinoma after margin-free resection on frozen section: an institutional perspective. Cureus.

[6] Mori T (2022). Involvement of the p53-p16/RB pathway control mechanism in early-stage carcinogenesis in head and neck squamous cell carcinoma. Pathol Int.

[7] Hashmi AA, Sajid A, Hussain M (2021). Mutant phenotype p53 immunohistochemical expression is associated with poor prognostic parameters and disease-free survival in triple-negative metaplastic breast carcinoma. Cureus.

[8] Hashmi AA, Hussain ZF, Hashmi SK (2018). Immunohistochemical over expression of p53 in head and neck squamous cell carcinoma: clinical and prognostic significance. BMC Res Notes.

[9] Blahak J, Zelinka J, Gumulec J (2020). HPV, protein p16 and squamous cell carcinoma of the oral cavity. Biomed Pap Med Fac Univ Palacky Olomouc Czech Repub.

[10] Hashmi AA, Younus N, Naz S (2020). p16 Immunohistochemical expression in head and neck squamous cell carcinoma: association with prognostic parameters. Cureus.

[11] Hashmi AA, Hussain ZF, Aijaz S (2018). Immunohistochemical expression of epidermal growth factor receptor (EGFR) in South Asian head and neck squamous cell carcinoma: association with various risk factors and clinico-pathologic and prognostic parameters. World J Surg Oncol.

[12] Palumbo C, Benvenuto M, Focaccetti C (2023). Recent findings on the impact of ErbB receptors status on prognosis and therapy of head and neck squamous cell carcinoma. Front Med (Lausanne).

[13] Hashmi AA, Hussain ZF, Irfan M (2018). Prognostic significance of epidermal growth factor receptor (EGFR) over expression in urothelial carcinoma of urinary bladder. BMC Urol.

[14] Verma J, Dhingra V, Srivastava S (2018). Evaluation of epidermal growth factor receptor expression by a new scoring system in head-and-neck squamous cell carcinoma and its association with various pathological prognostic factors. J Oral Maxillofac Pathol.

[15] Kakkar V, Sarin V, Chatterjee A (2022). Expression of cyclin-D1 and p53 as prognostic markers in treatment of oral squamous cell carcinoma. Indian J Otolaryngol Head Neck Surg.

[16] Szentkúti G, Dános K, Brauswetter D (2015). Correlations between prognosis and regional biomarker profiles in head and neck squamous cell carcinomas. Pathol Oncol Res.

[17] Kudo Y, Kitajima S, Ogawa I (2005). Down-regulation of Cdk inhibitor p27 in oral squamous cell carcinoma. Oral Oncol.

[18] Gao L, Gu W, Zheng J (2013). Clinicopathological and prognostic significance of p27 expression in oral squamous cell carcinoma: a meta-analysis. Int J Biol Markers.

